# Families' Experiences Living with Acquired Brain Injury: “Thinking Family”—A Nursing Pathway for Family-Centered Care

**DOI:** 10.1155/2020/8866534

**Published:** 2020-08-03

**Authors:** Jane Karpa, Wanda Chernomas, Kerstin Roger, Tuula Heinonen

**Affiliations:** ^1^University of Brandon, Winnipeg, Canada; ^2^College of Nursing, Rady Faculty of Health Sciences, University of Manitoba, Winnipeg, Canada; ^3^Community Health Sciences, University of Manitoba, Winnipeg, Canada; ^4^University of Manitoba, Faculty of Social Work, Winnipeg, Canada

## Abstract

The objective of this study was to examine families' experiences living with acquired brain injury (ABI) using a research approach that included *both* the affected individual family member and the family together as a family group. A narrative inquiry study, informed by the life-stage approach of Lieblich, Tuval-Mashiach, and Zilber, was used to obtain family stories. Families experiencing an ABI event were purposefully selected from different regions in a western Canadian province. Centered on the life stages of before the ABI event, now living with the ABI, and the future, thematic findings included: Families, a grounding force; Losses, individual and family; Family adaptive capacities; Experiences with the healthcare system-hospital to home; and A patchwork future-entering the unknown. Themes affirmed the significant impacts of ABI on individual and family members and acknowledged ABI as an ambiguous loss event. The findings also illuminated families' strengths and resiliencies in coping with living with ABI. The study results suggest by “*thinking family*” nurses can contribute towards a healthcare model that focuses on “family” as the central unit of care.

## 1. Introduction

“*Thinking family*” is a pivotal perspective for nurses' understanding and supporting individuals and families living with acquired brain injury (ABI). While the impact of ABI on individuals and families, and their corresponding sense of losses, has been well documented, generating knowledge and understanding of families' experiences of living with ABI has largely derived from examination of the subsystems within families—to either present their views of the family or provide their own perceptions of being in the family. Subsystems studied have included the individual with the ABI [1–8]; the caregiver or primary family carer [9–18], children [19–23], siblings [24–28], and the marital relationship [29–36]. Whilst the subsystem research has provided further understanding of individuals' experiences living with ABI, a family systems approach that studies the family in interaction with each other is limited. This approach purports that individuals cannot be understood in isolation; rather they need to be understood within the context of the “whole” family unit [37, 38]. Family members interact reciprocally; therefore, family members' perspectives are a result of the interplay between each other.

While there is growing interest in the experiences of the family as a whole; to date, only a limited number of qualitative studies have explored this area. Landau and Hissett [39] employed the family group as a unit of analysis to explore the relationship between loss of identity issues faced by the family member with a mild brain injury and the family's sense of loss of the member who is now different. Kean [40] collected data from family group interviews (12 adults, 12 children of various ages), to investigate families' experiences with critical illness in an intensive care setting. To obtain family perspectives of psychosocial transitions during the first 18 months post moderate to severe ABI, Whiffin et al. [41] interviewed nine non-injured family members from three families at three separate intervals post ABI and the data were analyzed on three levels: the individual, the family, and between family units. To determine the dynamics of relationships in families of patients with brain injury, Segev et al. [42], studied 10 family cases and completed separate individual interviews with the spouse and a family of origin member (i.e., father, mother, sibling or child). These studies have demonstrated, when research with families is informed by congruence with a systems perspective, new knowledge is acquired about the whole of families' experiences living with ABI.

Furthermore, understanding families' perspectives of the impact of ABI is important as family members continue to report they require enhanced understanding of their needs and want the family to be considered the primary unit of attention and care [43, 44]. The family-centered care paradigm, informed by principles of partnership and collaboration, has been garnering attention and evolving within health care over the past 10 to 15 years [45]. Yet, struggles to fully implement a family-centered practice model remain, as nurses and other healthcare practitioners, while considering families to be valuable contributors to treatment, continue to limit family involvement [46].

Informed by ambiguous loss theory, and employing a family research model that included *both* the individual family member and family members together as a group, the purpose of this study was to understand families' experiences with ABI; to explore the impact of ABI on families' attitudes, beliefs and identities; and to gain greater understanding of families' relational experiences to each other, their community, and healthcare practitioners for the purpose of supporting a family-centered model of care. The study results suggest by “*thinking family*” nurses can contribute to a healthcare model that focuses on “family” as the central unit of care.

## 2. Theorectical Background

This study was informed by ambiguous loss theory. This theory proposes a more systematic view of individuals and their families [47]. The concept of ambiguous loss is defined as a unique stressor situation in which there is an unclear loss resulting from not knowing whether a loved one is dead, alive, absent, or present [48]. There are two types of ambiguous loss: (1) a loved one is physically absent yet kept psychologically present, and (2) a loved one is physically present but psychologically absent [48]. A loved one who has disappeared in body (physically missing) is often kept psychologically present by family and community members, because the loss is not verified by evidence of death [47, 49]. This physical absence could be a result of war, terrorism, ethnic cleansing, genocide, kidnapping, and natural disasters. Conversely, an individual may be missing in mind (physically present yet be psychologically absent)—that is, emotionally or cognitively different [47, 49]. Examples of this type of ambiguous loss include people living with Alzheimer's disease, dementia, brain injury, AIDS, autism, depression, addiction, or other chronic mental or physical illnesses [47].

Ambiguous situations often are not tolerated well by people. Ambiguous loss is a stressor event and becomes more difficult and stressful the longer the ambiguous loss situation continues without resolution [47]. In either the physically absent or psychologically absent ambiguous state, the result may be unresolved grief as well as an uncertainty about who is in ‘the family'. This uncertainty about who is ‘in the family' is called boundary ambiguity. It is manifested when families experience role upheavals and disturbances in regular routines and family ritual practices. This study on families' experiences living with ABI incorporated the second type of ambiguous loss.

### 2.1. The Researcher

The researcher is an experienced mental health clinician with many years of professional clinical practice work with individuals and families affected by ABI. The researcher also experienced living with a family member diagnosed with an ABI. To mitigate bias and support trustworthiness, the following strategies were utilized: (1) triangulation of data; (2) rich thick description; (3) member checking; (4) audit trail documenting methods decisions; (5) reflexive journaling; and (6) discussing emerging themes with colleagues.

## 3. Methods

A narrative inquiry qualitative approach was utilized in this research project to capture family group stories about their experiences living with ABI. Narrative inquiry is a useful methodology for examining families affected by an ABI because of its ability to encapsulate how families make sense of their experiences living with ABI through the characteristics of meaning, relatedness, identity, and time. This is the first known study to incorporate narrative inquiry with family research and utilize the life-stage approach of Lieblich et al. [50].

### 3.1. Recruitment and Participants

Families were recruited from different regions of a province in Canada. Mutliplex criteria for both the individual and family members were considered. Criteria for the individual with the brain injury included medical stability; recovery from any acute medical conditions; no mechanical ventilation; living in the community; having English language communication skill capabilities; and ability to provide informed consent. A two-year postinjury criterion was established for the family members to secure family member participants who had passed the traumatic and episodic impact of the acute stage of the brain injury.

Consistent with the definition the family is who they believe themselves to be “a self-defined group of individuals” ([51], p.284), ‘family' consisted of at least the individual member with the ABI and up to a maximum of four other perceived family members. For the purpose of maintaining feasibility, a boundary was placed on the maximum number of family member participants. In accordance with beliefs of a family's right to self-refer, family members determined which family members participated. To help family members in their selection process, guidelines for selection were employed. These included persons who support, share a history and a future, and are committed and caring towards each other. Neither the individual member with the ABI nor the other family members needed to be residing within the same household. These guidelines were written permitting families to include friends as well as biological and legal family member participants. All individual and family participants were required to be a minimum of 18 years of age at the time this study was conducted.

Six families participated in this study (Table 1 insert near here). Narrative inquiry with families is unsuitable for a large number of participants as it involves a time commitment and close collaboration between participants and the researcher [52]. Recruitment was challenging, taking over one year to complete. Participants were recruited through ABI community support agencies.

All six participating families were unique, varying in structure, culture, and ages. Family structures included; (1) intact biological members (father and or mother and children); (2) blended families; and (3) friends as family members. One family self-identified as Aboriginal. Participant ages ranged from 23–67. Regarding the individual family members with the ABI, their brain injuries resulted from either nontraumatic or traumatic and were diagnosed within a range of moderate to severe. Of the six family member participants that had the brain injury, only one was male.

Potential bias may have been introduced in the recruitment process as family members may have chosen their members for participation based on family members' willingness to participate and level of connection.

### 3.2. Setting

The face to face interviews were held at a convenient location that protected the families' confidentiality. All family units chose to hold the interview in a family member's home. Within the setting environment, family members had full visibility of each through circular seating around a dining table or in the living room area.

### 3.3. Data Collection Procedure

Data collection was completed using several strategies. Transactional level data (data generated through discernable interactions amongst multiple family members) were collected through in-depth face to face interviews with individual family units; which included the individual with the ABI and other family members. Data were also collected by ethnographic methods including: family genogram; family group observation sociogram; family ecomaps; and field notes. Also, data were collected on how family members chose who would participate.

The interviews were framed in the context of no right or wrong responses as viewpoints by all participants were considered valid. During the interviews, questions were asked requiring family members to think how a missing family member would have responded. During the interviews, to initiate the process of narrative opportunities, the premise of Lieblich et al.'s [49] life story stages was used to help families structure their narratives around their experiences living with ABI. Families were asked to think about their life experiences with ABI as three life-stage chapters in a book about their family. The first chapter was about their family life before the ABI event, the second chapter is now—living with ABI, and chapter three concerns their future family life. Initial narrative conversational opening prompt such as “Tell me about a time in your family before the ABI event that reflects who you are,” “Tell me about a time after the brain injury that reflects who you are as a family now,” and “tell me about how you envision your family's future “were used. Conscious efforts were made to elicit responses from all family participants. Family members were provided with individual summaries of their intact statements pulled from the original transcripts for them to review.

The study was approved by the research ethics committees of the relevant Universities {Approval numbers: E2016 : 126 (HS20242); 22276}. Each family member participant provided informed consent. Privacy and confidentiality considerations were adhered to, including the deidentification of all transcripts and the use of pseudonyms in reporting results.

### 3.4. Data Analysis

Narrative analysis, while having aspects common with other case-centered approaches, relies on accounts that are analytically treated as whole units rather than fragmented into coded categories as evidenced in other qualitative approaches [53]. Methods of family level data analysis are not readily identified in the literature as most models have been designed with the individual in mind. For the study on families' experiences of living with ABI, analysis models developed by Boss and Carnes[49] and Riessman [53] were adapted for transactional level data analysis and processed through two layers.

First, each family unit transcript was analyzed separately by within case analysis, followed by an across-case analysis. Interpretive within case analysis was accomplished using a combination of cell classifications outlined by Boss and Carnes [49] for the purpose of analyzing different components of the narratives such as holistic content; repeated words or phrases; the plot; discrete stylistic or linguistic characteristics of defined units of the narrative; and emotions. Subsequent to the within-case analysis, the across-case analysis was accomplished using the thematic analysis approach delineated by Riessman [53].

Individual interpretive family synopses were generated from the within-case analysis reflecting global impressions of how each family made sense of their experiences living with ABI. Subsequent to the within case analysis, the across case analysis was accomplished using the thematic analysis approach delineated by Riessman [53] resulting in the development of overarching master themes or narratives. Only the thematic findings of the across case analysis are presented.

## 4. Findings

The master narrative themes were structured to replicate the chronological boundaries of the three life-stage chapters of the narrative interview process: (1) Before the ABI Event—*Families: a grounding force,* (2) Now Living with the ABI Event—a. *losses individual and family,* b. *family adaptive capacities and,* c. *experiences with the healthcare system: hospital and home, and,* (3) The Future—*A patchwork future, entering the unknown.*

### 4.1. Before the ABI Event—Families: A Grounding Force

This theme was set in the chronology of before the ABI event centered on the belief of connectedness and closeness and was associated with the bonding of its members, leading to a sense of belonging. These families' value of belonging underpinned their actions emotions and patterns of relating and was the grounding force that maintained their commitment to the “family unit.”

“We've always said to ourselves that we were a close family—so we make more effort to do things regularly, so the closeness is there “(Stetler family).

“You would have seen a family of two working parents with children quite involved in the community and their sports and so on coming and going. And weekends always brought us together. Work took us away from our family the odd day, but the weekends would *always bring us back together as a family. We were a close family*—*closeness would be presence*—*coming around and physically being with you.*” (Mercer Family)

Connected…interested and involved in one another's lives.” (Holder family)

### 4.2. Now Living with the ABI—A. Losses: Individual and Family

There are three major themes within the chronological segment of Now Living with the ABI. The theme of losses: individual and family addressed the range of complications experienced by the individual family member with the brain injury and the impact of these impediments on family members. Whether the original brain injury diagnosis was considered mild, moderate, or severe each, of the individual family members who were diagnosed with an acquired brain injury talked about experiences of being different after the ABI. Their individual experiences of difference were mirrored by their families' perceptions that their loved one was also not the same as before.

Individual family members with the ABI expressed the following:

It took many months before I could handle more than a conversation with one person at a time. I think it was probably months, I couldn't talk to you and look at you at the same time. I had to keep my eyes closed or looked down because my brain was overwhelmed with information. I couldn't have a radio on. I couldn't watch television. It was years before I could watch and I enjoy sports, say watch a football game, because there was just way too much going on. So I couldn't be part of social things. (Terri Holder)

I mean not working anymore, and not driving for the last many months. Anything that I was taking on personally was now taken on by somebody else. Not only that, taking care of me. I guess over the year, I've been dealing with anger, frustration, and lack of independence. You want to just be back to your old self. And I know it's never going to be the same. (Evelyn Cross)

Family members of the individuals with the ABI were also aware of the changes to their loved ones and talked about how they were or are continuing to be affected by these changes. The participating families acknowledged changes and differences to identity, relationships, social activities, and societal supports which had created an overarching sense of loss. For the Carter, Stetler, Holder, and Cross families, the suggested experiences of loss were about degrees of loss as the initial acute impairments experienced by their loved ones were now residual or were continuing to improve at the time of the family interviews. However, for the Wilson and Mercer families, their sense of loss was significant and permanent. May Wilson described “*When Marie had that accident, it was like we lost a family member. After the accident she was a different person. We have never really known what she would have been like before her accident…*” Frank and Margaret Mercer expressed “*The children…our grandchildren, they lost their mother.*” Families recognized other losses, experiencing social isolation as family friends disengaged and disappeared from their lives. Shelley Cross also understood a loss of societal support.

If it was just paralysis on one side, then people give the extra time or accommodate or hold open doors or whatever the case would be that way. And if they need physical support people support them. But it's the same thing as with a mental illness, when you don't see the situation, it's hard to empathize or understand what's difficult and different.

### 4.3. Now Living with the ABI—B. Family Adaptive Capacities

This second major theme revealed a powerful thread of strength and resiliency as families coped with the ABI event. The coping these families exhibited moved beyond a coping of subsisting or survival, rather their strength and resiliency were evidenced in these families' abilities to build capacity to effectively adapt to change. These families demonstrated the capacity to reorganize roles; self educate; and incorporate beliefs that further engendered acceptance and solidified their sense of belonging and identity as a family unit. These abilities showed the families to be responsive and consider the needs of all family members while retaining the similar mechanisms for family function and structure.

The participating families' capacity to reorganize roles was displayed in their ability to shift roles, specifically from child to parent, husband to caregiver, grandparent to parent, and friends as caregivers. Several of the participating adult child family members to varying degrees undertook aspects of the parental role by providing substantial support and encouragement to their parent and assisting with daily activities that would have previously been in the domain of their parent. Shelley Cross expressed the following:

I would say that my brother and I have more communication and contact. Like “have you talked to mom?” “Have you checked in?… I don't mind going grocery shopping. I did the Christmas shopping this year because the thought of (my mom) going to stores, waiting in lines, it's too much, too many tasks all at once…

For Mike Stetler, Brent Holder and Greg Cross their role as marital partner veered in the direction of more responsibility in taking care of their spouses; responding to and managing their illness needs. Greg Cross clearly acknowledged the transition to caregiver and its impact on him.

I am the caregiver at this point. And I'm okay with it. There was a time, when I literally had to be around all of the time. I basically, in a sense, you take your own life and you just sit it on a shelf somewhere and forget everything that you do. And now you're a caregiver for somebody. And you do it because you care. That's why it's a caregiver. But that wears on you after a while. And the little things that you want to do, that you always did, and you can't do anymore because they may be external pressures that she can't handle.

The role shift from grandparent back again to parent was encountered by Frank and Margaret Mercer. When Melanie had her ABI event, they needed to reclaim parental duties and functioning as Melanie's ability to parent was severely impaired. They expressed the following:

We were getting ready to retire. We were living in a condo and when we finally decided that we needed to become more fully involved with our grandchildren and our daughter, we walked away from our jobs basically. That's how we tried to assist Melanie by moving in with the family…we became the strong support for them. We didn't just become the grandparents; we also became the caregivers.

As well, family friends became caregivers. Mona and Macey, friends to the Holder family stated “As friends, we would give them whatever support they needed at any time. It was pretty much every day or twice a day.”

The onset of the ABI event was for all the participating families their first and only exposure to this diagnosis. Self education as a coping strategy was utilized by the participating families to build capacity to effectively adapt to change and manage living with the ABI. Several families educated themselves about brain injury; acquiring knowledge of what happened and developing understanding and awareness of what to expect and or anticipate. Shelley Cross articulated: “*I think as a family, when mom was in the hospital, we did things like send scholarly articles and research and we were trying to educate ourselves as fast as possible in that situation.*”

Beliefs are personal attitudes that allow for meanings to be considered as a basis of human emotions and actions. These families revealed mutually shared interpretations or beliefs about the brain injury event that aided them in effectively coping with living with ABI. Participating families expressed beliefs were prominently positive in nature. Whether characterized by faith-based language, ideas of living in the moment, or the commencement of another life path, these belief systems enabled effective adaptation to living with the ABI by reinforcing the families' bonds to each other; the grounding force behind their demonstrations of strength and resiliency.

From the perspective of the Carter family, the ABI event was a near death experience that provided Allen with a second chance to reevaluate his life and reconnect with his sons. Jeff named this belief a “Blessing.” The Wilson's family mantra of “*one day at a time*” and “*just do it*” attitude became more pronounced following the ABI event of their daughter Marie. “*One day at a time that's all you get. It's all you need. That's all we got…we just did it*.” Frank Mercer expressed the belief that the ABI event was a “gift”; “*But we get a gift. We get to spend a lot of time with our grandchildren. So, there are some fringe benefits from that too. We get to see them grow and not everybody gets to do that so.*” Frank also believed the ABI event was the start of a new family beginning because it “*changed everything*”.

### 4.4. Now Living with the ABI—C. Experiences with the Healthcare System: Hospital and Home

This third major theme explored the experiences families had or continue to have with the healthcare system from initial hospitalization to community treatment and supports. While families' experiences were described in this theme, this theme also revealed missing elements in their experiences and these omissions were interpreted. Overall, families' experiences of the healthcare system varied depending on the cause of the ABI, the severity of the ABI, the recovery process, and the degrees and kinds of service involvement (i.e., mental health services, community rehabilitation services, community support groups, and or insurance agencies {worker's compensation or vehicle insurance}). However, the similarity in families' experiences was the distinction between acute care and rehabilitation and community-based care. Family members spoke about the decrease in resource availability following acute care.

Frank Mercer stated the following:

When you get into recovery, that's when the healthcare system starts to deteriorate… where they haven't got time to spend time with the patient. Melanie with her injury, would maybe wander away. So, they had her tied to a wheelchair. And then because if she started to yell for things or whatever, they started to sedate her to slow her down. And that's when we became more involved as parents. So, Margaret would go 8 o'clock in the morning when she woke up and spend all day with her so they didn't have to sedate her. And I would go at 4 : 30 and stay till midnight until she went to sleep. And we did that for 4½ months as parents. That was just so that the healthcare system would not sedate her and drug her up. So, we were more involved as a family being there.

Families involved with insurance service providers considered themselves lucky because resource availability included financial support for additional rehabilitation and caregiving costs. However, these resources were also limited. The Mercer family had to advocate for additional funding and resources when insurance support was denied or ended. Margaret recounted:

You phone people. You gotta write. You go there, you go here. You get moved around and stuff like that. The difficulty with the doctors. Counselling for my grandchildren. ‘What about them?' Insurance simply said, well no, we don't cover the children, this is just for the client. So, then I made an effort, how many different places I called to try and get help for them. Couldn't do it.

Families also noted a reduction in resource availability in rural centers. May Wilson contended; “*But you get out to these smaller towns, and there's not as many supports or, or no supports out there.*” As they shared their experiences within the healthcare system, families touched on how healthcare professionals engaged with families. Mike Stetler communicated: “*the hospital is run really good. The surgeon actually showed us exactly what he did.*” May Wilson spoke about all of the appointments; “*Marie had a lot of appointments. I had to write every little thing down because she had so many appointments.*” And Mitchell Wilson followed up by saying; “*The insurance company would book appointments. They* wouldn't relay*that on to us and they would phone to confirm an appointment and May never had any knowledge of the appointment.*” Shelley Cross recounted a time when her mom was still in the hospital; the family requested that Evelyn be allowed to audio-record (using her phone) the doctors' conversations with her because she could not remember what they were telling her and therefore could not relate the information to her family. While some of the doctors allowed the recording, others did not. These descriptions give the impression healthcare professionals are primarily engaging with the individual in care.

On one level, the findings for this theme indicated these families experienced resource inadequacies within healthcare systems and healthcare professionals focused on the person in care. Missing from families' perspectives was the element of how families as a unit were cared for by the healthcare system. For families, their involvement with the healthcare system was in relation to the individual. Their focus was singularly centered on their loved one with the ABI or a family subunit; and their cognitive, physical, and emotional energies were directed towards trying to ensure that resources were made available to those individuals. Their experiences did not include family members talking about being part of the treatment process in which healthcare professionals collaborated with them and engaged with them as being experts on their own family members or asked about family needs.

### 4.5. The Future—A Patchwork Future: Entering the Unknown

This final master theme illustrated how living with the ABI continues to influence families' thoughts and feelings about their future. Families expressed a patchwork of thoughts: hope and optimism for continued recovery and successful progression through ongoing life stages as life carries on, while also conveying undercurrents of fear and worry about legalities of arranging for future care and supports and potential for occurrence of another ABI event. These findings suggest living with ABI is an undertaking that extends into the future, continuing to impact these families as they try to anticipate the unknown. However, no matter what their thoughts and feelings about the future these families continued to emanate their future together as a collective force.

“I think it's a progression of what it is right now. We're just moving with my dad to see what's happening. So, I guess we'll take it as we see it. But we'll always be there.” (Carter family)

“I don't know where Marie's going to end up. So, I guess we'll be here if Marie's here and you know we'll be helping her out as much as we can.” (Wilson family)

“We do have a document through the lawyer…Melanie will always need some assistance with living. The children are already getting a sense that they will hold the unit together and be with their parents and things like that. So, the future, it'll still go on. Family look after family and this is family.” (Mercer family)

## 5. Discussion

Predominantly, the literature has consistently reported ramifications of ABI on families as negative; with prolonged exposure to stress and strain and harmful effects on families' social, emotional, structural, and financial functioning; role changes; and challenges to core values and resources in families [14, 54–62]. Rather, the findings of this study offer a counterbalance to these bleak reports.

This study illuminated families' strengths and resiliency by demonstrating families have inherent competencies and adaptive capacities that help them to establish effective psychosocial coping and functioning while living with ABI. Instead of families experiencing increasing loss of identity issues [39], in this study, families' beliefs about their identity as a family unit were maintained and became more solidified while living with the ABI. These families described an increased sense of belonging and used their relationships as resources for each other, thereby having less functional difficulties [42]. While families in this study described stressful and challenging experiences, the role changes they recounted were not viewed as a loss that was negative and burdensome [41]; rather families demonstrated flexibility as they accepted the forced role changes and adapted. Establishing collective beliefs and meanings of the ABI situation helped these families tolerate the losses and adjust to the different circumstances. Participant families' demonstrations of strength and resiliency attest to their capabilities as carers and supporters of their loved ones with ABI.

In taking over the caregiving responsibilities for the members with the ABI, participating families in this study encountered issues with the healthcare system. Family members spoke about the decrease in resource availability following acute care. Their experiences with the healthcare system are affiliated with the literature reporting on unmet service and support needs throughout the continuum of care from hospital to home [62–67]. In addition, findings highlighted that families do not consider themselves to be in collaborative relationships with healthcare professionals. Compounding lack of collaborative relationships with the fact that families do not always know what they should ask for or expect [68] suggests the healthcare system is continuing to restrict involvement of the families in the planning, delivery, and evaluation of care [45, 46]. At the same time, research has identified families want to be involved in all aspects of care [43, 44]. Research has also shown healthcare providers are often hesitant to include family members, as families are seen as barriers in the patient-health clinician relationship [69].

These findings elucidated families' strengths, resiliencies, and expertise in living with ABI and also exposed relationship challenges between families and healthcare professionals. Findings suggest minimal supports exist for families impacted by ABI and nurses, and other healthcare professionals need to acknowledge and attend to the entire family system and not just the individual and primary care giver. In particular, the findings highlight the need for healthcare practitioners to continue to adopt practices informed by frameworks espousing strengths-based care and family-centered care. The prominence of the medical model has created a healthcare system focused on a systematic approach to diagnosis and treatment, reducing people's identities to a disease process, thereby distancing the relationships between individuals, families, and healthcare professionals [70]. The foundation of strengths-based care is the focus on persons' and families' strengths in order to promote care that empowers persons and families to take control of their own health and healing [70]. Based on the factors of respect, information sharing, participation, and collaboration [45], the essential ingredients of the family-centered care model are collaboration and partnership with the entire family for the purpose of planning, delivery, and evaluation of health care [69].

For nurses working with ABI individuals and families, the first practice step is to purposefully “*think family*” and then adopt intentional actions of knowing families' strengths and capabilities to collaborate with them throughout the stages of recovery and living with ABI [68]. Nurses are more effective collaborators when they generate greater understandings of family needs. Gaining knowledge about the person in the family who has the greatest influence on member health, family expectations, family decision making dynamics, and individual and family perspectives helps build effective partnerships and communication [68]. As with client-centered practice, the linkage between family-centered care and improved individual and family health outcomes is supported by the evidence [71, 72].

The study findings highlight the need for ongoing family research as these findings have only touched the surface of what can be learned from families who are coping in living with ABI. This study has exposed the need to continue researching the development of family centered care frameworks which recent articles within the ABI and loss literature have started [69, 73]. These findings when viewed through the paradigm of strengths-based care offer an alternative from preoccupation with what is going wrong and needs fixing to focusing on positives and what is going well [70]. A systematic review on strengths-based approaches working with families affected by progressive neurological illness revealed there is little evidence of the use of strengths-based approaches with this population [74]. To date, there is also minimal evidence of the strengths-based paradigm, theoretical or otherwise, being considered in conjunction with ABI and families.

## 6. Strengths and Limitations

The choice of narrative inquiry methodology and the a priori determined life chapters were significant as they explicated the characteristics of temporality, relatedness, meaning, and identity and helped in making narrative development less awkward. Temporality was affixed to the families' narratives in their lives before the ABI, now living with the ABI, and the potential future of living with the ABI. Relatedness was manifested by the families' acute sense of belonging to each other, which further adhered them to their identity as a ‘family'. These families' beliefs revealed the ways in which they interpreted their world in living with ABI and how they built shared meaning which strengthened their value of family.

Families' experiences of loss adhered to the definition of ambiguous loss as a unique stressor event in which a loved one is physically present but psychologically missing. Families' descriptions of their loved ones being ‘not the same', ‘different,' or ‘lost' substantiated the ambiguous loss definition. To varying degrees, these families continue to experience ambiguous loss because there is no resolution, as there is in a clear-cut death.

Research with families can be a rewarding yet intricate and complex process. Awareness of barriers to data collection arose as individuals may have been interested in participating, yet they did not want family members involved, or family did not want to be involved, or there were impediments in gathering family together for an interview. Recommendations for researchers interested in doing family research is to recruit widely and include longer recruitment time frames.

## 7. Conclusions

The exploration of families' experiences living with acquired brain injury affirmed that impacts of ABI are relational and revealed that while families contend with ambiguous loss, they have the capacities and competencies to affect their own healing processes. Through this study, it became apparent that living with ABI is a life process underscored by the need for nurses to incorporate relational thinking and practices that focus on getting to know families and collaborating with them on any potential needs and or supports. This study, by illuminating *the individual and family together*, can facilitate further development and implementation of family research across multiple health issues.

## Figures and Tables

**Table 1:** Family unit participant demographics.


*The Carter family:*
Allen—father, traumatic brain injury 17 years ago
David—oldest son
Jeff—younger son
*The Wilson family:*
May—mother
Mitchell—father
Ann—older daughter
Marie—younger daughter, traumatic brain injury eight years ago
Tradder (pseudonym)—family pet dog
*The Stetler family*
Debbie—wife and mother, nontraumatic brain injury two years ago
Mike—husband
Rob—younger son
Collen—step daughter
Trudy—sister to Debbie
*The Mercer family*
Frank—father
Margaret—mother
Melanie—daughter, traumatic brain injury 12 years ago
*The Holder family*
Terri—wife and mother, traumatic brain injury 10.5 years ago
Brent—husband
Matt—son
Mona—close family friend
Macey—close family friend
*The Cross family*
Evelyn—wife and mother; nontraumatic brain injury one and half years ago
Greg—husband and stepfather
Shelley—daughter
Curtis—son

## Data Availability

Underlying data from the results of this study reside with the first author.
